# Coexistence of Vertebral and Intervertebral Disc Changes in Low Back Pain Patients—In Depth Characterization with Same Day MRI and CT Discography

**DOI:** 10.3390/diagnostics13233528

**Published:** 2023-11-24

**Authors:** Hanna Hebelka, Alfred Erkmar, Helena Brisby, Kerstin Lagerstrand

**Affiliations:** 1 Institute of Clinical Sciences, Sahlgrenska Academy, University of Gothenburg, 413 45 Gothenburg, Sweden; alfred.erkmar@vgregion.se (A.E.); helena.brisby@vgregion.se (H.B.); kerstin.lagerstrand@vgregion.se (K.L.); 2 Department of Radiology, Sahlgrenska University Hospital, 413 45 Gothenburg, Sweden; 3 Department of Orthopaedics, Sahlgrenska University Hospital, 413 45 Gothenburg, Sweden; 4 Department of Medical Physics and Biomedical Engineering, Sahlgrenska University Hospital, 413 45 Gothenburg, Sweden

**Keywords:** disc degeneration, vertebral endplate, modic changes, degenerative disc disease, annular fissure

## Abstract

The aim of this study was to investigate to what extent annular fissures, vertebral and endplate changes, and Modic changes (MCs), coexist in low back pain (LBP) patients by using multiple imaging modalities. Sixty-two LBP patients (mean age 45 years, range 24–63, 53% men) were examined with same-day CT-discography and MRI. Intervertebral discs punctured for discography (*n* = 204) were evaluated on MRI [Pfirrmann grade, High-Intensity Zone (HIZ)] and on CT-discograms [Modified Dallas Discogram Score (DDS)]. DDS≥ 1, i.e., disc fissures involving the outer annulus were further digitomized into delimitable fissuring (<50% of annulus affected) or non-delimitable annular fissuring. Using both MRI and CT, adjacent vertebrae and endplates were assessed for MC, vertebral sclerosis, and a modified endplate defect score (EPS). In 194 discs the contrast agent was adequately injected during discography, of which 160 (83%) displayed outer annular fissures, with 91 (47%) of the latter being delimitable fissures. Most discs with delimitable fissures were moderately degenerated; 68% Pfirrmann grade ≤3, 71% EPS ≤ 2, and 12% displayed MC. The majority (76%) of MCs were associated with advanced adjacent disc degeneration; 84% Pfirrmann grade ≥4, 76% with non-delimitable annular fissuring, 59% EPS≥ 4, and 34% EPS of 3. A total 95 HIZ (47%) were found, of which 54 had delimitable fissuring, while the remainder displayed non-delimitable fissuring. Vertebral sclerosis was commonly observed (26%), both with MCs (73%) and without MCs (27%), and not specifically linked to MC type 3. A total of 97% of segments with vertebral sclerosis displayed outer annular fissures. These findings were significant (0.046 > *p* > 0.0001), except between HIZ and adjacent sclerosis (*p* = 0.303). To conclude, the present study confirmed a close interplay between the disc and adjacent vertebra and endplates. The fact that a majority of discs with delimitable annular fissures did not coexist with pronounced endplate changes and/or MCs, however, supports the theory that disc fissuring is an early event in the degenerative cascade. This was further supported by the fact that MCs were strongly linked to extensive disc fissuring and to advanced endplate damage. Further, vertebral sclerosis was common also in vertebra without MCs and strongly associated to annular fissuring, indicating that sclerosis is a previously underestimated feature of a general degenerative process.

## 1. Introduction

Low back pain (LBP) is a very common condition that affects people of all ages and is one of the leading causes of disability worldwide [1]. LBP can have a significant impact on both the affected person’s quality of life as well as socioeconomically [1], which is why it is important to drive research within the area forward, to improve diagnostics and outcome for these patients.

When the underlying cause of pain cannot be identified, or when there is no specific structural abnormality causing the pain, the condition is referred to as nonspecific low back pain (NSLBP), which accounts for approximately 85–90% of all cases of LBP [2,3]. While the exact cause of NSLBP often is unclear, it is known to be associated with various degenerative spinal changes, such as disc degeneration, facet joint arthrosis, annular fissures, Modic changes (MCs), and endplate changes [4,5]. Even if degenerative spine changes are more common in NSLBP patients, degeneration is part of the natural ageing process and common also in asymptomatic individuals [4], which is why the relationship is complex, and more research is needed to fully understand the mechanisms involved in pain experienced by NSLBP patients.

As NSLBP likely is multifactorial [3,5,6], and it is appealing to investigate relationships between multiple potential factors. MCs, annular fissures, and endplate changes are all commonly found in patients with NSLBP, and they may coexist in the same individual [7,8,9,10]. While the relationship between these entities is not entirely clear, there is some evidence that they may be interrelated [6,8,9,10,11,12]. One simplified theory is that endplate changes may lead to the development of MCs and annular fissures by altering the nutrient supply to the vertebrae, or by causing mechanical stress on the vertebral body [6,13]. Another theory is that annular fissures are the initiating event, eliciting inflammatory mediators, which in turn affect the endplate and eventually also the subchondral bone [6,13]. Likely both scenarios can occur. Either way, the association between endplate changes, MCs, and annular fissures is complex and requires further investigation, preferably with multimodal imaging and adequate reference methods, to fully understand the association between these entities. 

Chronic LBP is a common symptom of a heterogeneous group of causative conditions [14]. Enhanced research within imaging diagnostics, to enable more accurate phenotyping of spinal pain conditions, may improve patient selection and enable early, targeted treatment intervention. Detailed characterization of multiple morphological spinal features, each reported to be associated with LBP, will deepen the knowledge regarding if and how coexistence between these features appears [14]. Most in vivo imaging studies investigating possible relationships are often limited by either a lack of reference method for annular fissures, i.e., discography, or do not use multiple imaging modalities; or if so, are not performed the same day, and therefore may not have the possibility to characterize each entity with enough detail.

The aim of this study was to investigate to what extent vertebral and endplate changes, MCs, and annular fissures coexist in LBP patients by using multiple imaging modalities [Computed Tomography (CT)/Magnetic Resonance Imaging (MRI)/discograms], performed the same day, for improved detailed characterization of the respective entity.

## 2. Materials and Methods

A total of 62 NSLBP patients (mean age 45 years, range 24–63, 53% men), previously enrolled in comparative (same-day) discography-MRI studies (1.5 T, sagittal T2/T1-weighted images covering L1-S1), were included [15,16]. Patients included in the original trial were patients referred for preoperative lumbar discography during a 3-year period. All had at least 6 month history of LBP which had failed conservative therapy and was severe enough to consider the patients for surgery. Patients with allergies to contrast media or an inability to undergo MRI were not eligible. All included patients underwent first an MRI followed by a discography and a subsequent CT, in accordance with clinical routine at that time. For each patient, all three examinations were performed during the same day.

MRI was performed on a 1.5 Tesla MRI scanner (Siemens Magnetom Symphony Maestro Class, Erlangen, Germany) including 4 mm T1- (TR 541 ms/TE 11 ms) and T2-weighted (TR 4.000 ms/TE 124 ms) sagittal slices, and 4- mm T2 (TR 5.000–6.970 ms/TE 114–116 ms) axial images.

All IVDs that had been injected with contrast for discography purpose were included and evaluated on MRI by a senior radiologist (>15 years) according to Pfirrmann Classification and if HIZ existed or not. Based on a modified Dallas Discogram Description [17], the IVDs were further classified on CT, post discography, in the axial plane according to if they had annular fissures extending to the outer third of the annulus (grade 1), extending beyond the outer third, i.e., leakage beyond the IVD (grade 2), or if no annular fissures could be seen involving the outer annulus (grade 0) [18,19]. IVDs with DDS ≥ 1 were further digitized according to whether the fissure was delimitable or not, with the former defined as a clearly delimitable fissure, or several such fissures, but where <50% of the outer annulus is involved. Hence, a non-delimitable fissure was defined as a diffusely degenerated IVD where >50% of the outer annulus is affected by fissures.

**Figure 1:**
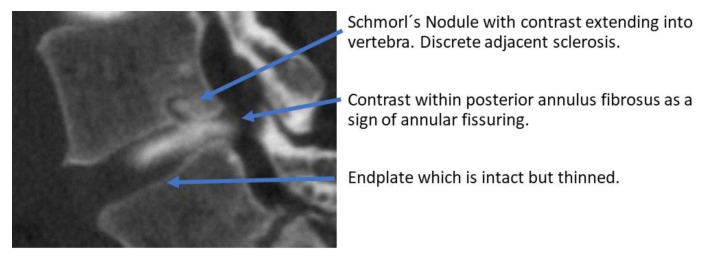
Example of CT-discogram with contrast extending into adjacent vertebrae.

Vertebrae and endplates adjacent to the discograms were evaluated according to the existence of MC (MRI), vertebral sclerosis on CT, and whether contrast from the discogram extended into the adjacent vertebrae (CT). Contrast extending to adjacent vertebra was evaluated as not existing (0) or existing (1), with the latter defined as IVD contrast on CT extending into the vertebra or clearly through the endplate (Figure 1).

The study was conducted according to the Declaration of Helsinki. Oral and written informed consent was obtained from all participants (Dnr 366-07 and Dnr 2022-03018-02)

The endplates were further classified according to a slightly modified endplate defect score [20] according to a combined appearance on CT/MRI (Table 1). MCs, evaluated solely on MRI appearance, were classified as type 1, type 2, type 3, or mixed type 1/2, type 2/3, type 1/3, with ≥20% of the MCs affected by a certain type to be classified as mixed. Further, to be classified as a MC, it needed to be visualized on at least two consecutive sagittal images and affect 25% of the vertebra on either anterior–posterior direction or craniocaudal direction.

Since each IVD is bordered by a superior and inferior vertebra/endplate, each vertebra was divided into two equally large units, with each vertebral unit/endplate unit evaluated separately. This was done in order to be able to investigate relations between an IVD and both the adjacent vertebrae and endplates, i.e., both the superior unit of the adjacent inferior vertebra and the inferior unit of the adjacent superior vertebrae. Any findings in adjacent units were fused, i.e., if one adjacent vertebra did not show any findings while the other adjacent unit did for example display MC type 3, the “total” finding in that vertebral segments was MC type 3, or if one unit displayed mixed MC and the other displayed MC type 1, the “total” vertebral segment was classified as the “highest” score/classification, i.e., in this case MC type 1.

### 2.1. Reliability Measures 

To assess inter-observer and intra-observer agreement for MC, Pfirrmann grade, and EPS, the radiologist repeated the evaluation on 30 segments after 3 months, as did a medical student trained within the methodology. Intra- and inter-observer agreement regarding fissure extension on discograms has been reported with high agreement (κ = 0.96–1.0) within the same research group [18]. Also, the agreement in assessment of HIZ has previously been reported as high, with a Chronbach alpha of 0.76 [21].

### 2.2. Statistics

The data were analyzed using version 9.4 of the SAS System, mainly with descriptive statistics presented as number and percent. Correlation analyses of categorical data are presented as number and percent, and significant covariation is regarded as *p* < 0.05. The *p*-value in the bar charts presented shows whether there is a significant correlation between two categorical variables. The bars visually describe the association.

Fishers exact test was used to test 2 × 2 comparisons, and the Mantel Haenszel Chi Square test was used for ordered categorical data. 

The intra- and inter-observer reliability of the Pfirrmann grading, MC, sclerosis, and EPS was determined with Cohen’s kappa coefficient (κ). The coefficients were interpreted according to Landis and Koch [18]. Kappa values between 0.0 and 0.2 represent slight agreement, values between 0.21 and 0.40 represent fair agreement, values between 0.41 and 0.60 represent moderate agreement, 0.61 to 0.80 represent substantial agreement, and values exceeding 0.81 represent almost perfect agreement. 

## 3. Results

### 3.1. Distribution of Morphological Changes within the Cohort

In total, 204 IVDs were included, and consequently 204 adjacent vertebral/endplate segments (204 adjacent superior units and 204 adjacent inferior units, fused to represent 204 adjacent vertebral/endplate segments) were evaluated. Since 10 IVDs failed to be adequately injected (annular injection and/or other technical failure to adequately evaluate the discogram) a total of 194 CT discograms were included in the analysis. Distribution data of Pfirrmann grading, HIZ, and annular fissures according to DDS, among the 204 IVDs, are displayed in Table 2, as are distribution of EPS and MCs.

### 3.2. Intervertebral Disc Changes Discs in Relation to Endplate Score and Modic Changes

**Figure 2:**
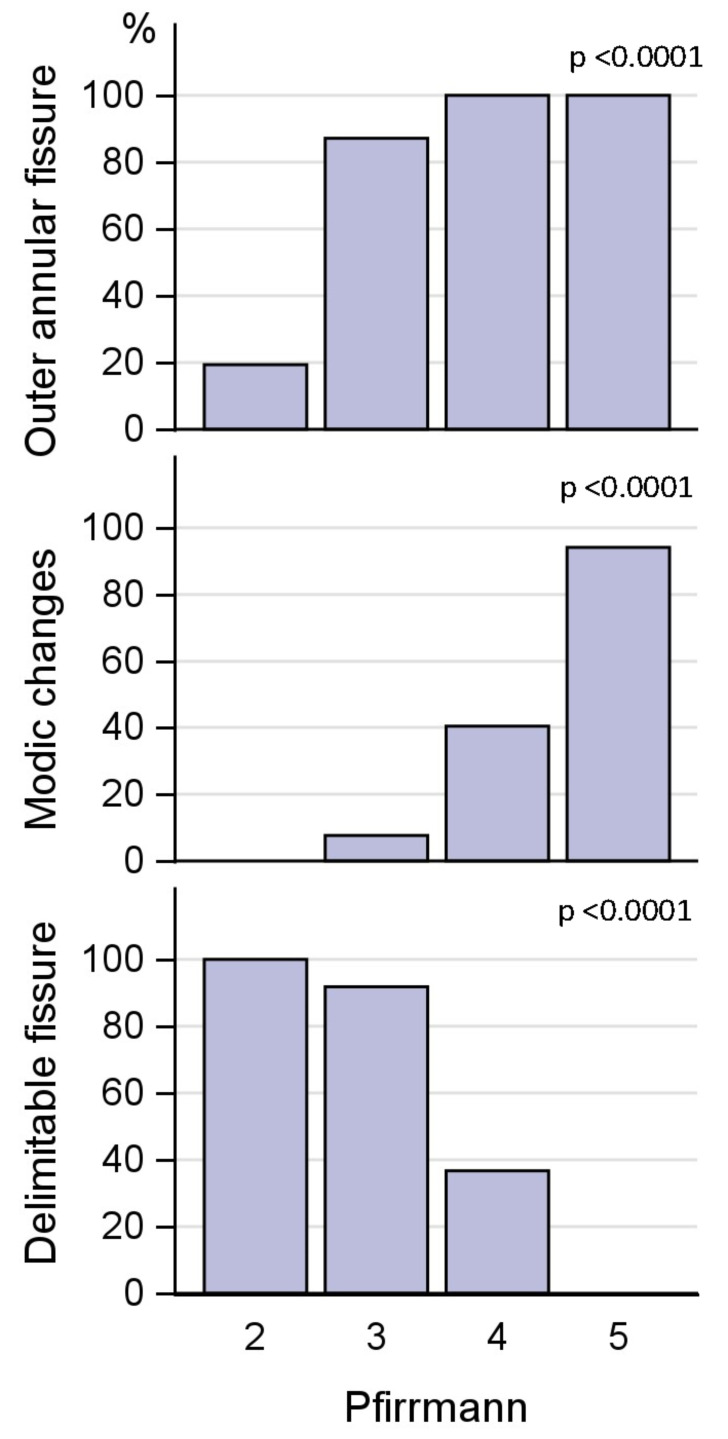
Covariation of Pfirrmann grade and annular fissuring.

**Figure 3:**
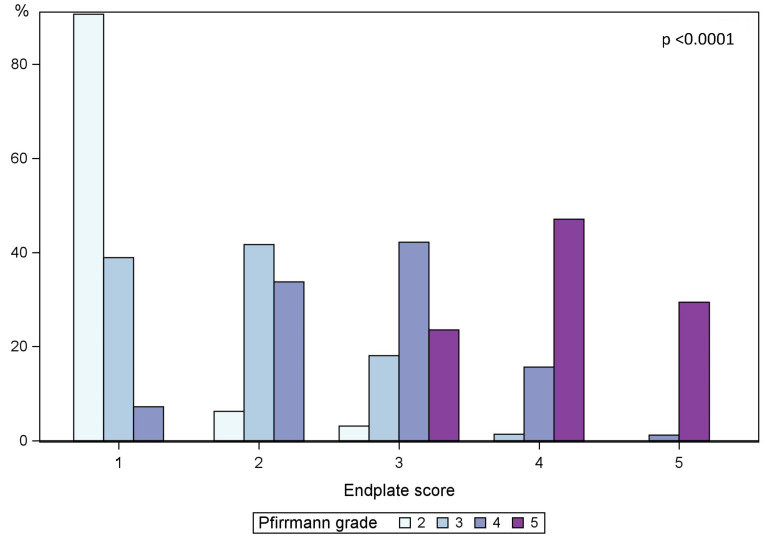
Covariation of endplate score and Pfirrmann Grade. Pfirrmann grade distribution stratified for endplate score as a percent.

Of the 160 IVDs with outer annular fissures, 91 were classified as having a delimitable fissure (Table 2). The majority of IVDs with delimitable fissures were moderately degenerated; of the 91 IVDs with delimitable fissures, 6% had Pfirrmann grade 2, 62% displayed Pfirrmann grade 3, and 32% grade 4. All IVDs with Pfirrmann grades 4 and 5 had outer annular fissures, as did 84% of Pfirrmann grade 3 IVDs, and 20% of Pfirrmann grade 2 IVDs. Pfirrmann grade was significantly associated with outer annular fissures and delimitable fissures (both *p* < 0.0001) (Figure 2). The association between Pfirrmann grade and EPS is displayed in Figure 3.

**Table 2:** Distribution of morphological changes in intervertebral discs, endplates, and vertebrae.

Number of Intervertebral Discs Affected (%)							Number of Endplate Segments Affected (%)		Number of Vertebral Units Affected (%)		
MRI n = 204				CT discograms n = 194			MRI/CT combined n = 204		MRI n = 408		
Pfirrmann Grade		HIZ		Outer Annular Fissure		Delimitable Fissures	Endplate Defect Score		Modic Change MC		Sclerosis on CT Stratified by MC
									MC type 1	10 (2.5)	5 (50)
1	0	yes	95 (46.6)	yes = DDS ≥ 1	160 (82.5)	91 (46.9)	1	49 (24.0)	MC type 2	66 (16.1)	41 (62.1)
2	32 (15.7)	no	109 (53.4)	no = DDS 0	34 (17.5)		2	65 (31.9)	MC type 3	3 (0.7)	3 (100)
3	72 (35.3)						3	51 (25.0)	MC mix 1&2	15 (3.7)	13 (86.7)
4	83 (40.7)						4	31 (15.2)	MC mix 2&3	9 (2.2)	8 (88.9)
5	17 (8.3)						5	8 (3.9)	MC mix 1&3	8 (2.0)	7 (87.5)
									no MC	297 (72.8)	28 (9.4)

**Figure 4:**
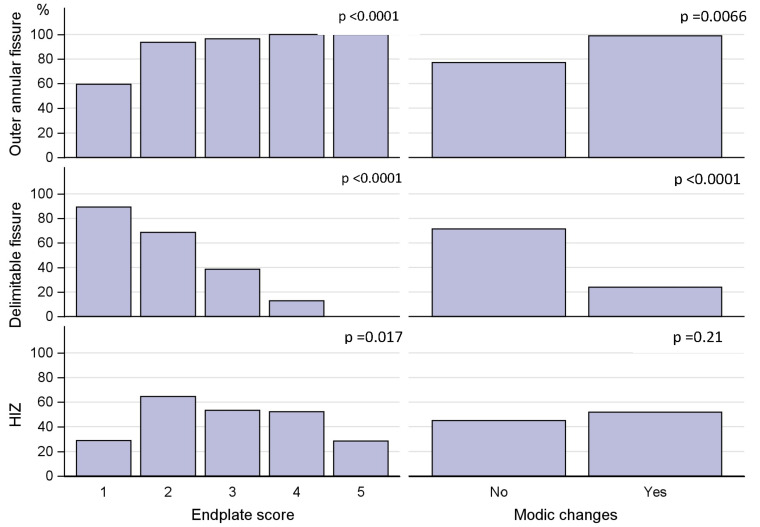
Covariation between endplate defect score, Modic changes, and annular fissuring.

A majority (71%) of IVDs with delimitable fissures displayed an EPS of 2 or less, 23% displayed an EPS of 3 while only 6% had an EPS of 4. The association between both EPS and outer annular fissures and EPS and delimitable fissures was significant (*p* < 0.0001) (Figure 4).

If an IVD displayed delimitable fissures, it was not common with MCs in that segment. Only 11 out of 91 IVDs (12%) with delimitable fissures showed MCs, and of all segments with MCs, 76% of adjacent IVDs were diffusely degenerated, i.e., >50% of annulus affected by fissures, and only 23% had delimitable fissures (Figure 4). The association between MCs and outer annular fissuring was significant (*p* < 0.01).

Ninety-five HIZs (47%) were found, of which 54 had delimitable fissure on the discogram, while the remaining IVDs had >50% of the annulus affected by fissures. The sensitivity for HIZs as a marker for outer annular fissures (*n* = 160) was 59%, and the sensitivity for HIZs as a marker for delimitable fissures (*n* = 91) was also only 59%. A majority of IVDs with HIZs displayed only moderate affection of the endplate. Of the 95 IVDs with HIZs, 53% displayed an EPS of 2 or less, 26% an EPS of 3, 19% an EPS of 4, and 2% an EPS of 5 (Figure 4). 

### 3.3. Coexistence of Modic Changes and Endplate Score

Sixty-two segments (30%) displayed some type of MC. Distribution and type of MC for all 408 vertebral units are shown in Table 2. Of all segments with MC, 59% displayed extensive EP damage, i.e., an EPS of 4–5 in adjacent endplates, and 34% displayed an EPS of 3. This association between segments with MC (any type) and EPS was significant (*p* < 0.0001) (Figure 4).

Although rare, MCs were also found in segments with only slight EP damage. One-hundred-fourteen segments had an EPS of 1–2, of which only four segments (3.5%) displayed MCs. Of 204 segments, 39 (19%) displayed extensive EP damage, i.e., an EPS of 4–5, of which only 2 segments (5%) did not display any MC at all. A significant association was further found between MC and existence of both outer annular fissures and EPS (*p* < 0.0001).

### 3.4. Sclerosis in Relation to Modic Changes and Annular Fissures

An in depth analysis was performed on each vertebral unit (*n* = 408), along with the existence of MCs and type of MC, to elucidate if, and to what extent, sclerosis existed also in units without MCs or in MCs without type 3. Vertebral sclerosis was a common morphological feature also without MCs and not specifically found in MCs involving type 3 (Table 1). There were 104 vertebral units with sclerosis, stratified according to lumbar level accordingly: L2 (*n* = 4), L3 (*n* = 3), L4 (*n* = 14), L5 (*n* = 30) and S1 (*n* = 53). The distribution of MCs in relation to sclerosis is displayed in Table 1.

Of the 65 segments with vertebral sclerosis, 97% had outer annular fissures (*p* = 0.0016). Among the 91 IVDs with delimitable fissures, 74% did not display adjacent sclerosis. Any significant association between HIZ and adjacent sclerosis was not found (*p* = 0.303) 

### 3.5. Contrast Extending into the Vertebrae

**Figure 5:**
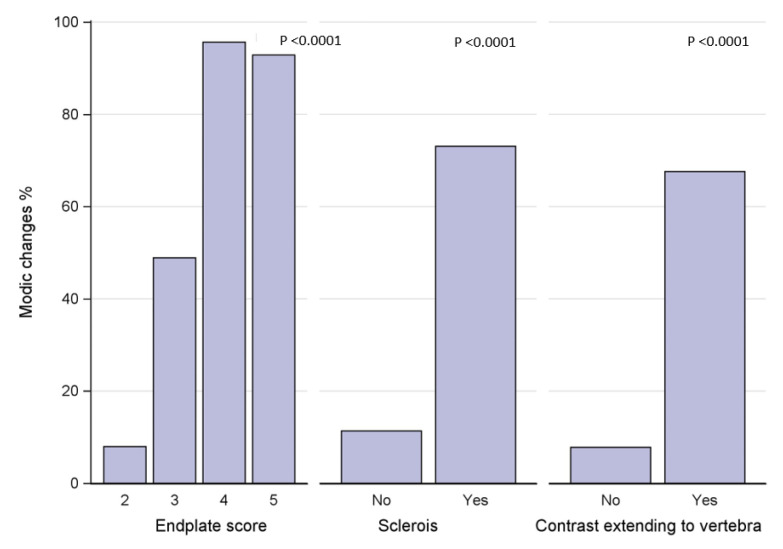
Endplate defect score, vertebral sclerosis, and vertebral contrast extension in relation to Modic changes.

Contrast extending into the vertebra, an apparent sign of a disrupted barrier between the IVD and vertebrae, was found in 57 segments. Of discograms with contrast extending into the vertebra, 95% also had outer annular fissures, i.e., DDS ≥1, in the axial plane, an association that was significant (*p* = 0.008). Of the segments with contrast in the vertebra, 61% displayed sclerosis and 63% MCs, associations that both were significant (both *p* < 0.0001) (Figure 5). Stratified per vertebral unit, 108 units displayed contrast extending through the endplate. Of these, 55% were categorized having an EPS of 3 and 43 % an EPS of 4–5, and 68% displayed adjacent MCs. 

### 3.6. Reliability Measures

The intra-observer reliability measures for MC (k = 0.7; *p* < 0.001), Pfirrmann grading (k = 0.9; *p* < 0.001), sclerosis (k = 1.0; *p* < 0.001), and EPS (k = 0.8; *p* < 0.001) were all substantial or almost perfect. Inter-observer reliability measures för Pfirrmann grading (k= (0.6; *p* < 0.001) and EPS (k = 0.5; *p* < 0.001) were moderate, and for MC (k = 0.7 (*p* < 0.001) and sclerosis (k= 0.8, *p* < 0.001) substantial.

## 4. Discussion

In this study, in which a detailed characterization of morphological features in spinal segments was investigated using multiple imaging modalities performed during the same day, a close interplay between the IVD and adjacent vertebra and endplate was confirmed [11,12,22,23]. The novelty in this work was the finding that IVDs with delimitable annular fissures seldom coexisted with endplate changes and/or MCs. Also, even if HIZ on an MRI is a marker for disrupted annulus fibrosus, it was shown to have low sensitivity for delimitable disc fissures. Additionally, it was confirmed that HIZ also has poor sensitivity for whether an IVD has fissures involving the outer annulus or not, which previously had been reported [24].

MCs were strongly linked to extensive fissuring of the IVD and to advanced EP damage. Another main finding was that vertebral sclerosis is a common finding, seen also in vertebrae without MC and not specific for MC type 3. Extension of contrast into the vertebra, as a sign of an apparent damaged barrier between IVD and vertebra, was strongly linked to both advanced endplate damage and vertebral sclerosis.

In healthy spines, only the outer annulus fibrosis is innervated [25]. Nerve ingrowth, through annular fissures, into the inner layer of the disc, can occur [26]. Annular fissures thus have potential to be a source of pain and are of special interest to study more in detail. The majority of IVDs with delimitable fissures displayed only minor endplate and vertebral changes, strengthening the argument that these fissures likely are an initiating event [27], as they represent an early imaging manifestation of the degenerative cascade, rather than a more pronounced such manifestation. This finding aligns with the concept that subtle changes in the annulus fibrosus precede subsequent nuclear degeneration [27,28] and then a more pronounced degeneration [29], highlighting the importance of identifying these early indicators of degeneration. HIZ, representing tears in the outer annulus fibrosus [30,31], has been regarded as one such early degenerative sign. In concordance with previous studies, this study, using the gold standard for fissure characterization, shows that even though HIZ is a sign of annular injury, it is a marker with poor sensitivity for whether an IVD has an outer annular fissure [24]. Moreover, HIZ had also a low sensitivity in identifying delimitable fissures. HIZ, composed by vascularized granulation tissue, has been proposed as a potential diagnostic marker for symptomatic internal disc disruption, i.e., discogenic NSLBP [32,33]. It has been proposed that disrupted annular fibers allow inflammatory mediators to irritate nearby nerves, leading to pain [32]. HIZ’s clinical relevance as a reliable marker for NSLBP, and its role in guiding treatment decisions, remain, however, a subject of research and discussion [30,34,35]. The current results show that HIZ as a marker for a specific morphological fissure type is limited and underscore the complexity of interpreting HIZ and its relevance in diagnosing certain disc conditions accurately. This likely also explains the divergent research results regarding HIZ and NSLBP. Technical imaging advancements can aid in improving fissure detection non-invasively [24,36] and future studies are encouraged to use such techniques to better elucidate how NSLBP and annular fissure types are associated.

The fact that neither MC etiopathology nor their association to NSLBP have been fully elucidated highlights the importance of improving the knowledge of MCs. MCs have been suggested to be a result of either inflammation, infection, or mechanical damage, or a combination of two, or all three [37,38,39,40]. This study cannot draw any conclusions regarding causality, however current results indicate an initiating IVD related component, since early signs of disc deterioration are often detected without vertebral changes. This is in opposition to whether MCs are detected, where there is almost always advanced IVD fissuring and advanced endplate damage. The strong association between advanced endplate damage and both MCs and outer annular fissures amplifies the clinical value of detecting also discrete MCs, as these indicate more advanced deterioration in that spinal segment. 

Vertebral sclerosis was a common finding in all types of MCs. Although this has been reported in other studies [41,42,43], the current study challenges the conventional assumption that, foremost, MC type 3 are composed by vertebral sclerosis. Sclerosis was additionally commonly observed in segments without any MC, displaying the complexity of MC lesions and the shortcomings of the present classification system as well as the shortcomings with conventional MRI, where endplate and vertebral lesions can be overlooked [43]. Rajasekaran et al. concluded in their MRI study of over 8000 vertebral segments that conventional MC classification was flawed by the fact that over 580 segments needed reclassification, showing previously undetected oedema when adding STIR sequence, also displaying the shortcomings with the “classic MC classification system” [23]. Sclerosis, i.e., increased bone density, a result of increased osteoblast activity and reactive bone remodeling [42], can reflect an active inflammatory process as a response to underlying insult in the IVD/endplate/vertebrae. For example, spinal segments with advanced IVD degeneration, frequently associated with apparent MC and endplate changes, have been associated with increased uptake on SPECT/CT in adjacent vertebrae, in opposition to segments with only slight IVD degeneration [42]. Since inflammatory mediators promote bone formation, these image findings strengthen the arguments for a concomitant, quite extensive, inflammatory process associated with MCs, irrespective of the initiating etiopathology. Although the clinical relevance of vertebral sclerosis remains unclear, the current results highlight the need for nuanced interpretation of vertebral sclerosis, especially within a research context, to better understand the clinical relevance of vertebral sclerosis in relation to other findings of degenerative spinal pathology.

IVD contrast extending into the vertebra, or at least through the endplate, a marker of an apparent damaged barrier between the IVD and vertebra, was strongly associated with both advanced endplate damage and sclerosis. Apparent MRI features of such a disrupted barrier are Schmorl’s nodules, which are indentations or herniations of IVD material into adjacent vertebral bodies [44]. Schmorl´s nodules are also associated with inflammation in surrounding bone [45]. While they have also been suggested as a potential contributor to NSLBP, their direct association with pain is debated [44,45]. With the current study setup, we had the opportunity also to study less obvious communications between IVD and vertebrae in more detail. Some theories suggest that Schmorl’s nodules, and thus indirectly also less pronounced disrupted IVD–vertebral barrier, might contribute to LBP by causing inflammation, sensitizing nerves, or inducing structural changes in the spine [44,45]. This study cannot draw any conclusions in relation to pain, but the results display that the presence of disrupted IVD–endplate interface is strongly related to more severe degenerative processes affecting both the IVD and the vertebral body itself.

One may ask why it is important to drive the research forward within the area of NSLBP and spinal morphological features. As discussed herein, NSLBP is influenced by a complex interplay of multiple contributing factors in the vertebrae, endplate, and IVD. Degeneration and inflammatory processes, nerve affection, and alterations in nutrient supply likely all play roles in the development and persistence of unspecific LBP [23,35,44,46,47]. Hence, it is important to study all these factors in detail, but also combined. Previous as well as present studies are hampered by limited diagnostic ability and subjectivity during image interpretation. However, a strength of the current study on clinical LBP patients is the use of multiple imaging modalities, performed the same day, including the use of gold standard discography for fissure characterization. Thereby, this study contributes to a deeper understanding of the relationships between various morphological features within the spine, the limitations of certain markers, and early degenerative signs. 

In recent years, new and improved diagnostic tools have emerged in the field of LBP diagnostics. Machine learning algorithms, predictive modelling, and advanced image analysis, which all utilize advanced computational techniques, reduce the subjectivity in the image interpretation and can thereby enhance early detection, with the potential to improve the accuracy and efficiency of diagnosing and managing LBP [48,49,50]. It is likely such techniques will further aid in the understanding of the interplay between spinal structures and their relation to NSLBP.

## 5. Conclusions

Present findings confirmed a close interplay between the IVD and adjacent vertebra and endplate. While conclusions regarding causality cannot be reached, the study strengthens the hypothesis that degeneration generally is initiated in the IVD, as early degenerative IVD signs were found to frequently exists without endplate and/or vertebral affection.

This was further supported by the fact that MCs were strongly linked to extensive disc fissuring and to advanced endplate damage. These findings highlights that it is unreasonable to look at a single marker in the search for features linked to pain, and that a multiparametric approach is necessary. Finally, it should be noted that HIZ is a marker for annular disruption but a very non-specific one. Minor endplate defects and MCs indicate a more pronounced deteriorating process in that segment. Also, vertebral sclerosis is a common morphological feature also without MCs and not specifically found in MC type 3. 

## Figures and Tables

**Table 1:** Modified Endplate Defect Score.

Endplate Score		Combined Apperance on CT/MRI		
1	2	3	4	5
normal	focal disc contact/maintained contour	defects > 25%	defects up to 50%	extensive erosions/total destruction

## Data Availability

Data can be obtained upon request from the corresponding author.

## References

[1] Mokdad A.H., Mensah G.A., Krish V., Glenn S.D., Miller-Petrie M.K., Lopez A.D., Murray C.J.L. (2017). Global, regional, and national incidence, prevalence, and years lived with disability for 328 diseases and injuries for 195 countries, 1990–2016: A systematic analysis for the Global Burden of Disease Study 2016. Lancet.

[2] Koes B.W., van Tulder M.W., Thomas S. (2006). Diagnosis and treatment of low back pain. BMJ.

[3] Chiarotto A., Koes B.W. (2022). Nonspecific low back pain. N. Engl. J. Med..

[4] Brinjikji W., Diehn F.E., Jarvik J.G., Carr C.M., Kallmes D.F., Murad M.H., Luetmer P.H. (2015). MRI Findings of Disc Degeneration are More Prevalent in Adults with Low Back Pain than in Asymptomatic Controls: A Systematic Review and Meta-Analysis. Am. J. Neuroradiol..

[5] Luoma K., Vehmas T., Kerttula L., Grönblad M., Rinne E. (2016). Chronic low back pain in relation to Modic changes, bony endplate lesions, and disc degeneration in a prospective MRI study. Eur. Spine J..

[6] Adams M.A., Dolan P. (2012). Intervertebral disc degeneration: Evidence for two distinct phenotypes. J. Anat..

[7] Wang Y., Videman T., Battié M.C. (2012). ISSLS prize winner: Lumbar vertebral endplate lesions: Associations with disc degeneration and back pain history. Spine.

[8] Albert H.B., Briggs A.M., Kent P., Byrhagen A., Hansen C., Kjaergaard K. (2011). The prevalence of MRI-defined spinal pathoanatomies and their association with Modic changes in individuals seeking care for low back pain. Eur. Spine J..

[9] Lagerstrand K., Brisby H., Hebelka H. (2021). Associations between high-intensity zones, endplate, and Modic changes and their effect on T2-mapping with and without spinal load. J. Orthop. Res..

[10] Moser M., Amini D.A., Sanchez L.A., Oezel L., Haffer H., Muellner M., Zhu J., Carrino J.A., Shue J., Sama A.A. (2023). The association between vertebral endplate defects, subchondral bone marrow changes, and lumbar intervertebral disc degeneration: A retrospective, 3-year longitudinal study. Eur. Spine J..

[11] Sahin B., Akkaya E. (2022). Modic changes and its association with other MRI phenotypes in east Anatolian low back pain patients. Br. J. Neurosurg..

[12] Zehra U., Cheung J.P.Y., Bow C., Lu W., Samartzis D. (2019). Multidimensional vertebral endplate defects are associated with disc degeneration, modic changes, facet joint abnormalities, and pain. J. Orthop. Res..

[13] Adams M.A., Adams M.A., Lama P., Zehra U., Dolan P. (2015). Why do some intervertebral discs degenerate, when others (in the same spine) do not?. Clin. Anat..

[14] Conger A., Smuck M., Truumees E., Lotz J.C., DePalma M.J., McCormick Z.L. (2022). Vertebrogenic Pain: A Paradigm Shift in Diagnosis and Treatment of Axial Low Back Pain. Pain Med..

[15] Hebelka H., Brisby H., Hansson T. (2014). Comparison between pain at discography and morphological disc changes at axial loaded MRI in patients with low back pain. Eur. Spine J..

[16] Hebelka H., Hansson T. (2013). Erratum to: HIZ’s relation to axial load and low back pain: Investigated with axial loaded MRI and pressure controlled discography. Eur. Spine J..

[17] Sachs B.L., Vanharanta H., Spivey M.A., Guyer R.D., Videman T., Rashbaum R.F., Johnson R.G., Hochschuler S.H., Mooney V. (1987). Dallas Discogram Description a New Classification of CT/Discography in Low-back Disorders. Spine.

[18] Torén L., Lagerstrand K., Waldenberg C.M.P., Brisby H., Hebelka H. (2020). MRI During Spinal Loading Reveals Intervertebral Disc Behavior Corresponding to Discogram Findings of Annular Fissures and Pain Provocation. Spine.

[19] Eriksson S., Waldenberg C.M., Torén L., Grimby-Ekman A., Brisby H., Hebelka H., Lagerstrand K. (2021). Texture Analysis of Magnetic Resonance Images Enables Phenotyping of Potentially Painful Annular Fissures. Spine.

[20] Rajasekaran S., Venkatadass K., Naresh Babu J., Ganesh K., Shetty A.P. (2008). Pharmacological enhancement of disc diffusion and differentiation of healthy, ageing and degenerated discs: Results from in-vivo serial post-contrast MRI studies in 365 human lumbar discs. Eur. Spine J..

[21] Hebelka H., Gunterberg V., Lagerstrand K., Brisby H. (2023). Clinical outcome and MRI appearance in a group of chronic low back pain patients more than 10 years after discography evaluation and consideration for surgery. BMC Musculoskelet. Disord..

[22] Applebaum A., Nessim A., Cho W. (2022). Modic change: An emerging complication in the aging population. Clin. Spine Surg..

[23] Rajasekaran S., Bt P., Murugan C., Mengesha M.G., Easwaran M., Naik A.S., Ks S.V.A., Kanna R.M., Shetty A.P. (2023). The disc-endplate-bone-marrow complex classification: Progress in our understanding of Modic vertebral endplate changes and their clinical relevance. Spine J..

[24] Waldenberg C., Eriksson S., Brisby H., Hebelka H., Lagerstrand K.M. (2022). Detection of Imperceptible Intervertebral Disc Fissures in Conventional MRI—An AI Strategy for Improved Diagnostics. J. Clin. Med..

[25] Ohtori S., Inoue G., Miyagi M., Takahashi K. (2015). Pathomechanisms of discogenic low back pain in humans and animal models. Spine J..

[26] Freemont A., Peacock T., Goupille P., Hoyland J., O’Brien J., Jayson M. (1997). Nerve ingrowth into diseased intervertebral disc in chronic back pain. Lancet.

[27] Sharma A., Pilgram T., Wippold F. (2009). Association between Annular Tears and Disk Degeneration: A Longitudinal Study. Am. J. Neuroradiol..

[28] Vernon-Roberts B., Moore R.J., Fraser R.D. (2007). The natural history of age-related disc degeneration: The pathology and sequelae of tears. Spine.

[29] Isa I.L.M., Teoh S.L., Nor N.H.M., Mokhtar S.A. (2022). Discogenic Low Back Pain: Anatomy, Pathophysiology and Treatments of Intervertebral Disc Degeneration. Int. J. Mol. Sci..

[30] Ito M., Incorvaia K.M., Yu S.F., Fredrickson B.E., Yuan H.A., Rosenbaum A.E. (1998). Predictive Signs of Discogenic Lumbar Pain on Magnetic Resonance Imaging with Discography Correlation. Spine.

[31] Yu S.W., Sether L.A., Ho P.S., Wagner M., Haughton V.M. (1988). Tears of the anulus fibrosus: Correlation between MR and pathologic findings in cadavers. Am. J. Neuroradiol..

[32] Peng B., Hou S., Wu W., Zhang C., Yang Y. (2006). The pathogenesis and clinical significance of a high-intensity zone (HIZ) of lumbar intervertebral disc on MR imaging in the patient with discogenic low back pain. Eur. Spine J..

[33] Schellhas K.P., Pollei S.R., Gundry R.C., Heithoff K. (1996). B Lumbar disc high-intensity zone: Correlation of magnetic resonance imaging and discography. Spine.

[34] Carragee E.J., Paragioudakis S.J., Khurana S. (2000). Lumbar High-Intensity Zone and Discography in Subjects Without Low Back Problems. Spine.

[35] Teraguchi M., Yim R., Cheung J.P.-Y., Samartzis D. (2018). The association of high-intensity zones on MRI and low back pain: A systematic review. Scoliosis Spinal Disord..

[36] Waldenberg C., Hebelka H., Brisby H., Lagerstrand K.M. (2019). Differences in IVD characteristics between low back pain patients and controls associated with HIZ as revealed with quantitative MRI. PLoS ONE.

[37] Viswanathan V.K., Shetty A.P., Rajasekaran S. (2020). Modic changes—An evidence-based, narrative review on its patho-physiology, clinical significance and role in chronic low back pain. J. Clin. Orthop. Trauma.

[38] Mera Y., Teraguchi M., Hashizume H., Oka H., Muraki S., Akune T., Kawaguchi H., Nakamura K., Tamai H., Tanaka S. (2020). Association between types of Modic changes in the lumbar region and low back pain in a large cohort: The Wakayama spine study. Eur. Spine J..

[39] Fields A.J., Battié M.C., Herzog R.J., Jarvik J.G., Krug R., Link T.M., Lotz J.C., O’Neill C.W., Sharma A. (2019). ISSLS Degenerative Spinal Phenotypes Group Measuring and reporting of vertebral endplate bone marrow lesions as seen on MRI (Modic changes): Recommendations from the ISSLS Degenerative Spinal Phenotypes Group. Eur. Spine J..

[40] Dudli S., Liebenberg E., Magnitsky S., Miller S., Demir-Deviren S., Lotz J.C. (2016). *Propionibacterium acnes* infected intervertebral discs cause vertebral bone marrow lesions consistent with Modic changes. J. Orthop. Res..

[41] Zhu J., Wu H., Chen Y., Liu J., Shan Z., Fan S., Zhao F. (2021). The correlation between the change of Hounsfield units value and Modic changes in the lumbar vertebral endplate. BMC Musculoskelet. Disord..

[42] Van den Wyngaert T. (2023). Degenerative Spine: Osteophytosis–Endplate Changes. Clinical Atlas of Bone SPECT/CT.

[43] Rajasekaran S., Pushpa B.T., Soundararajan D.C.R., Anand K.S.S.V., Murugan C., Nedunchelian M., Kanna R.M., Shetty A.P., Tangavel C., Muthurajan R. (2022). Are Modic changes ‘Primary infective endplatitis’?—Insights from multimodal imaging of non-specific low back pain patients and development of a radiological ‘Endplate infection probability score’. Eur. Spine J..

[44] Azzouzi H., Ichchou L. (2022). Schmorl’s nodes: Demystification road of endplate defects—A critical review. Spine Deform..

[45] Takahashi K., Miyazaki T., Ohnari H., Takino T., Tomita K. (1995). Schmorl’s nodes and low-back pain: Analysis of magnetic resonance imaging findings in symptomatic and asymptomatic individuals. Eur. Spine J..

[46] Urban J.P., Fairbank J.C. (2020). Current perspectives on the role of biomechanical loading and genetics in development of disc degeneration and low back pain; a narrative review. J. Biomech..

[47] Maher C., Underwood M., Buchbinder R. (2017). Non-specific low back pain. Lancet.

[48] Lamichhane B., Jayasekera D., Jakes R., Glasser M.F., Zhang J., Yang C., Grimes D., Frank T.L., Ray W.Z., Leuthardt E.C. (2021). Multi-modal biomarkers of low back pain: A machine learning approach. NeuroImage Clin..

[49] Waldenberg C., Brisby H., Hebelka H., Lagerstrand K.M. (2023). Associations between Vertebral Localized Contrast Changes and Adjacent Annular Fissures in Patients with Low Back Pain: A Radiomics Approach. J. Clin. Med..

[50] Lagerstrand K., Hebelka H., Brisby H. (2022). Identification of potentially painful disc fissures in magnetic resonance images using machine-learning modelling. Eur. Spine J..

